# Proposal of De Novo Antigen Test for COVID-19: Ultrasensitive Detection of Spike Proteins of SARS-CoV-2

**DOI:** 10.3390/diagnostics10080594

**Published:** 2020-08-14

**Authors:** Yuta Kyosei, Mayuri Namba, Sou Yamura, Rikiya Takeuchi, Noriko Aoki, Kazunari Nakaishi, Satoshi Watabe, Etsuro Ito

**Affiliations:** 1 Department of Biology, Waseda University, 2-2 Wakamatsucho, Shinjuku, Tokyo 162-8480, Japan; yuta.18-baseball@asagi.waseda.jp (Y.K.); myrnmb@asagi.waseda.jp (M.N.); souyamura@toki.waseda.jp (S.Y.); 2 Research and Development Department, TAUNS Laboratories, Inc., 245-1 Doniwa, Shimizu, Sunto, Shizuoka 411-0903, Japan; r_takeuchi@tauns.co.jp (R.T.); n_aoki@tauns.co.jp (N.A.); s_watabe@tauns.co.jp (S.W.); 3 Quality Headquarters, TAUNS Laboratories, Inc., 761-1 Kamishima, Izunokuni, Shizuoka 410-2325, Japan; k_nakaishi@tauns.co.jp; 4 Waseda Research Institute for Science and Engineering, Waseda University, 3-4-1 Okubo, Shinjuku, Tokyo 169-8555, Japan; 5 Graduate Institute of Medicine, Kaohsiung Medical University, No. 100 Shiquan 1st Rd., Sanmin, Kaohsiung 80756, Taiwan

**Keywords:** COVID-19, SARS-CoV-2, spike protein, thio-NAD cycling, ultrasensitive ELISA

## Abstract

Polymerase chain reaction (PCR)-based antigen tests are technically difficult, time-consuming, and expensive, and may produce false negative results requiring follow-up confirmation with computed tomography. The global coronavirus disease 2019 (COVID-19) pandemic has increased the demand for accurate, easy-to-use, rapid, and cost-effective antigen tests for clinical application. We propose a de novo antigen test for diagnosing COVID-19 using the combination of sandwich enzyme-linked immunosorbent assay and thio-nicotinamide adenine dinucleotide (thio-NAD) cycling. Our test takes advantage of the spike proteins specific to the severe acute respiratory syndrome coronavirus 2 (SARS-CoV-2) virus. The limit of detection of our test was 2.3 × 10^−18^ moles/assay. If the virus has ~25 spike proteins on its surface, our method should detect on the order of 10^−20^ moles of virus/assay, corresponding to ~10^4^ copies of the virus RNA/assay. The detection sensitivity approaches that of PCR-based assays because the average virus RNA load used for PCR-based assays is ~10^5^ copies per oro- or naso-pharyngeal swab specimen. To our knowledge, this is the first ultrasensitive antigen test for SARS-CoV-2 spike proteins that can be performed with an easy-to-use microplate reader. Sufficient sensitivity can be achieved within 10 min of thio-NAD cycling. Our antigen test allows for rapid, cost-effective, specific, ultrasensitive, and simultaneous multiple measurements of SARS-CoV-2, and has broad application for the diagnosis for COVID-19.

## 1. Introduction

The world is currently combating a new viral outbreak that originated in China in 2019. The virus was named ‘severe acute respiratory syndrome coronavirus 2 (SARS-CoV-2)’ by the International Committee on Taxonomy of Viruses [1], and the World Health Organization (WHO) named the illness resulting from this virus ‘coronavirus disease 2019 (COVID-19)’ [2]. Some diagnostic tools have been proposed, including nucleic acid testing, computed tomography (CT), and others [3]. Nucleic acid testing is mainly based on a real-time polymerase chain reaction (PCR) method, and to overcome potentially false negative results of PCR, CT scans are often used for clinically diagnosing COVID-19 [4]. These methods can only be used in core hospitals, however, and are not available in many general clinics. Furthermore, PCR-based tests are technically difficult to perform, time-consuming, and expensive, and, as mentioned above, may provide false negative results [5]. An easy-to-use, rapid, cost-effective antigen test for accurately diagnosing COVID-19 is therefore urgently needed. Several antigen and antibody tests are being developed, and some kits have been commercialized [3,6].

On April 8, 2020, the WHO stated that it did not “recommend the use of antigen-detecting rapid diagnostic tests for patient care, although research into their performance and potential diagnostic utility is highly encouraged” [7]. There are 2 main reasons for this WHO judgement: (1) The main weakness of antigen tests is low detection sensitivity, which results in many false negative responses. (2) The assay must have very high detection specificity for accurate diagnosis. That is, if the antibodies used in the assay recognize antigens of viruses other than SARS-CoV-2, false positive responses would result. In fact, an important article published in *Science* on May 22, 2020, concluded that although coronavirus antigen tests are quick and cheap, they are too often wrong [8]. These 2 criticisms motivated us to develop a new antigen test with high detection sensitivity that uses anti-spike protein antibodies to confer high specificity in testing for the SARS-CoV-2 virus.

Genomic sequencing of SARS-CoV-2 revealed high homology with SARS-related bat viruses belonging to the family *Betacoronaviridae* [9]. This single-stranded RNA virus was isolated by the Victorian Infectious Disease Research Laboratory in Australia [10] and various other groups [11,12]. The 3′ terminus of the genome encodes 4 major structural proteins, including spike surface glycoprotein, small envelope protein, matrix protein, and nucleocapsid protein, as well as accessory proteins. The spike protein facilitates binding to the host cell receptor through angiotensin-converting enzyme 2 (ACE2) as the main receptor [13].

In the present study, we propose a de novo antigen diagnostic test for COVID-19, in which the spike proteins of SARS-CoV-2 are detected at the attomole level. This method is performed by combining a sandwich enzyme-linked immunosorbent assay (ELISA) and thio-nicotinamide adenine dinucleotide (thio-NAD) cycling. By targeting the spike proteins, we increased the test specificity for the SARS-CoV-2 virus.

## 2. Materials and Methods

**Figure 1:**
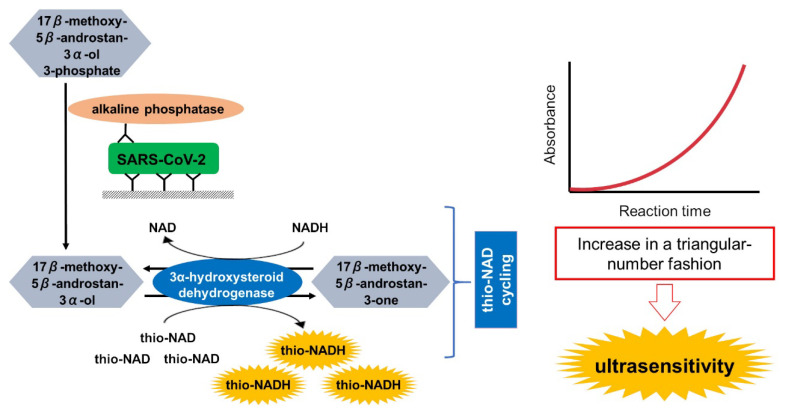
Principle of the ultrasensitive detection of SARS-CoV-2. The SARS-CoV-2 spike proteins were measured by a sandwich ELISA coupled with thio-NAD cycling using alkaline phosphatase, androsterone derivatives, and 3α-hydroxysteroid dehydrogenase (3α-HSD) and its coenzymes (NADH and thio-NAD). During this cycling reaction, thio-NADH accumulated in a triangular-number fashion. Accumulated thio-NADH was measured directly at an absorbance of 405 nm without interference from the other cofactors.

A sandwich ELISA coupled with thio-NAD cycling was originally developed by Watabe and Ito (Figure 1) [14]. The sandwich method used a primary and secondary antibody for the spike proteins of SARS-CoV-2 (i.e., antigen). The primary antibody was the COVID-19 recombinant human antibody of clone AM038105 (Cat No. 91339; Active Motif, Carlsbad, CA, USA), and the secondary antibody was AM002414 (Cat No. 91349; Active Motif) [15]. The antigen was spike protein (S1) of SARS-CoV-2 (human cell source, 78.3 kDa, Cat No. Z03501; GenScript USA, Piscataway, NJ, USA). For the control experiments, we used the spike protein of SARS-CoV, which comprises the ectodomain, containing 3 critical elements (i.e., S1, S2 and receptor binding domain). This was a kind gift from Yoshimasa Takahashi and Tadaki Suzuki (National Institute of Infectious Diseases, Tokyo, Japan). The secondary antibody was conjugated to alkaline phosphatase (ALP, EC. 3.1.3.1; Cat No. 03359123001; Roche, Mannheim, Germany) by using an Alkaline Phosphate Labeling Kit-SH (Dojindo Molecular Technologies, Kumamoto, Japan). As the primary substrate, we used 17β-methoxy-5β-androstan-3α-ol 3-phosphate [16,17]. For the thio-NAD cycling, 3α-hydroxysteroid dehydrogenase (3α-HSD, EC. 1.1.1.50, a kind gift from TAUNS, Shizuoka, Japan) was used. The 3α-HSD cofactors (i.e., thio-NAD and NADH) were purchased from Oriental Yeast (Cat No. 44101900 and 44320900, respectively; Tokyo, Japan). During the cycling reaction, thio-NADH accumulated in a triangular-number fashion, and the accumulated thio-NADH was measured directly at an absorbance of 405 nm without any interference from the other cofactors [18,19].

The experimental protocol was as follows [20,21,22]. A 100-μL solution of primary antibody, adjusted to 10 μg/mL in 50 mM Na_2_CO_3_ (pH 9.6), was added to microplate wells and incubated at room temperature for 1 h. The microplates were incubated with 1% bovine serum albumin (BSA) in Tris-buffered saline (TBS) at room temperature for 1 h. Spike protein (100 µL, i.e., antigen) was added to each well and incubated at room temperature for 1 h with shaking. The antigen samples were diluted with TBS containing 0.1% BSA. A 100-μL solution of secondary antibody, conjugated with ALP and adjusted to 33 ng/mL in TBS including 0.05% Tween 20 and 0.1% BSA, was then added to the wells and incubated at room temperature for 1 h with shaking. To amplify the ELISA signals, 100 μL of thio-NAD cycling solution was added to the wells. The thio-NAD cycling solution contained 1.0 mM NADH, 3.0 mM thio-NAD, 0.1 mM 17β-methoxy-5β-androstan-3α-ol 3-phosphate, and 30 U/mL 3α-HSD in 100 mM Tris-HCl (pH 9.0). The detectable signal (i.e., thio-NADH) was amplified in a triangular-number manner during the cycling reaction within a short time, and measured with a microplate reader at 405 nm. The 405-nm signals were normalized to those at 660 nm.

The experimental data were obtained by subtracting the mean value of the blank signals from each of the corresponding measured datapoints. The limit of detection (LOD) was estimated from the mean of the blanks, the standard deviation of the blanks, and a confidence factor of 3. The limit of quantitation (LOQ) was estimated by the same method used to estimate the LOD, but with a confidence factor of 10. The coefficients of variation (CVs) were obtained for spike protein antigen in the examinations of intra-assay and inter-assay reproducibility.

## 3. Results

**Figure 2:**
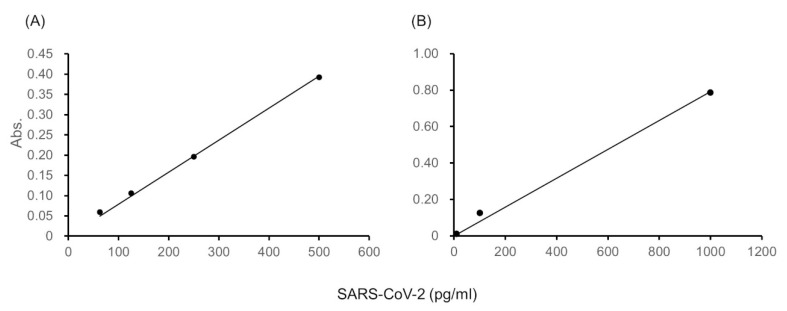
Linear calibration curves obtained from the absorbance. (**A**) Cycling reaction time of 60 min. This curve is expressed as *y* = 8.0 × 10^−4^*x*, *R*^2^ = 1.00 in the range of 63–500 pg/mL. (**B**) Cycling reaction time of 60 min. This curve is expressed as *y* = 8.0 × 10^−4^*x*, *R*^2^ = 1.00 in the range of 10–1000 pg/mL.

The ultrasensitive ELISA produced linear calibration curves for SARS-CoV-2 spike proteins as the antigen in the range of 10–2000 pg/mL (Figure 2). For example, the linear calibration curve that was obtained from the blank-subtracted absorbance of thio-NADH at 60 min of thio-NAD cycling for SARS-CoV-2 spike proteins in the range of 63–500 pg/mL could be expressed as *y* = 8.0 × 10^−4^*x*, *R*^2^ = 1.00 (Figure 2A), and that for SARS-CoV-2 spike proteins in the range of 10–1000 pg/mL as *y* = 8.0 × 10^−4^*x*, *R*^2^ = 1.00 (Figure 2B). In this case, the LOD of SARS-CoV-2 spike proteins was 2.3 × 10^−18^ moles/assay for the range of 63–500 pg/mL (Figure 2A), and the LOD was 2.4 × 10^−18^ moles/assay for the range of 10–1000 pg/mL (Figure 2B). The LOQ of SARS-CoV-2 spike proteins was 7.5 × 10^−18^ moles/assay for the range of 63 - 500 pg/mL (Figure 2A), and the LOQ was 8.0 × 10^−18^ moles/assay for the range of 10–1000 pg/mL (Figure 2B). Thus, the ultrasensitive ELISA detected SARS-CoV-2 spike proteins at the attomole level. Because the molecular mass of the antigen was 78.3 kDa and the assay volume was 100 μL, the LOD and LOQ correspond to 1.8 and 5.9 pg/mL, respectively.

**Figure 3:**
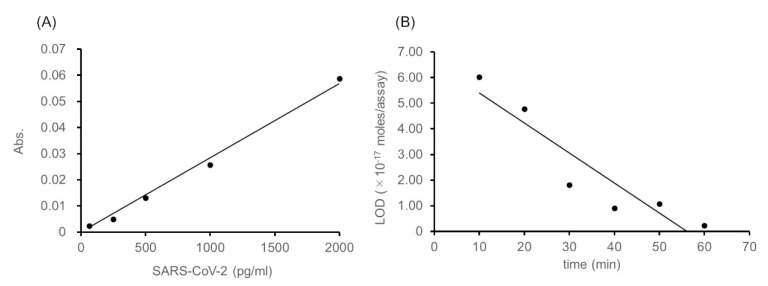
Linear calibration curve obtained from the absorbance at 10 min of thio-NAD cycling and the relation between the period of thio-NAD cycling and the LOD for SARS-CoV-2 spikes. (**A**) Cycling reaction time of 10 min. This linear calibration curve is expressed as *y* = 3.0 × 10^−5^*x*, *R*^2^ = 1.00 in the range of 63–2000 pg/mL. (**B**) The relation between the period of thio-NAD cycling and the LOD for SARS-CoV-2 spikes. This relation is expressed as *y* = −1.0 × 10^−18^*x* + 7.0 × 10^−17^, *R*^2^ = 0.86 in the range of 10–60 min of thio-NAD cycling.

We then attempted to detect SARS-CoV-2 spike proteins with 10 min of thio-NAD cycling. The resulting linear calibration curve was *y* = 3.0 × 10^−5^*x*, *R*^2^ = 1.00 (Figure 3A). The LOD was 6.0 × 10^−17^ moles/assay and the LOQ was 2.0 × 10^−16^ moles/assay. These values correspond to 47 and 157 pg/mL, respectively. We evaluated the relation between the period of thio-NAD cycling and the LOD for SARS-CoV-2 protein spikes (Figure 3B). This relation is expressed as *y* = −1.0 × 10^−18^*x* + 7.0 × 10^−17^, *R*^2^ = 0.86 in the range of 10–60 min of thio-NAD cycling, and thus the correlation was confirmed.

We then examined potential cross reactions between our SARS-CoV-2 detection system and another coronavirus, SARS-CoV. As described above, the spike proteins of SARS-CoV used as a control antigen included only the ectodomain, without the transmembrane region and cytoplasmic region. When both SARS-CoV-2 and SARS-CoV were examined at concentrations of 1000 pg/mL with the 60-min cycling time, the absorbance, from which the blank level was subtracted, was 0.79 ± 0.06 Abs and 0.06 ± 0.16 Abs (mean ± SD, *n* = 3 each; *p* = 0.004 by Student *t*-test), respectively. Thus, our SARS-CoV-2 detection system distinguished SARS-CoV-2 from SARS-CoV.

The intra-assay and inter-assay reproducibility results were as follows. In the intra-assay reproducibility, when 31, 125, and 500 pg/mL of spike protein antigen was used, the CVs obtained from 3 datapoints were 4.3%, 6.4%, and 1.2%, respectively. In the inter-assay reproducibility, when 3 different experimenters obtained the data using 1000 pg/mL, the CV was 6.6%, and when 3 experiments were performed on 3 different days by 1 experimenter, the CV was 7.6% (data not shown).

## 4. Discussion

We succeeded in detecting the spike proteins of SARS-CoV-2 at the 10^−18^ moles/assay level. In other words, if 1 spike protein is located on 1 virus, 10^6^ viruses can be detected per assay. We can assume, however, that the number of spike proteins located on the virus surface is approximately 25 [23]. Thus, our system can detect SARS-CoV-2 on the order of 10^−20^ moles/assay (i.e., 10^4^ viruses/assay). This value (i.e., 10^4^ RNA copies/assay) is noteworthy because previous studies on the diagnosis of COVID-19 by PCR-based methods using oro- and naso-pharyngeal specimens obtained from patients with suspected COVID-19 reported that the mean virus RNA load was 6.8 × 10^5^ copies per swab [24]. Another study reported that the detection results (i.e., threshold cycle values) of real-time PCR was 1.4 × 10^6^ copies/mL for nasal swabs, which is estimated to be 4.2 × 10^6^ copies/swab, because the specimen per swab was diluted with 3 mL of, for example, Universal Transport Medium (COPAN Diagnostics, Inc. Murrieta, CA, USA) [25]. Thus, these findings indicate that our antigen detection sensitivity approximates that of the PCR-based assays.

In June 2020, a chemiluminescent enzyme immunoassay (CLEIA) for SARS-CoV-2 was launched in Japan [26]. CLEIA requires a special instrument called the LUMIPULSE (Fujirebio, Tokyo, Japan). The cut-off value of the CLEIA assay (1.34 pg/mL) is almost the same as the LOD of our colorimetric system (1.8 pg/mL). This CLEIA assay detects nucleocapsid proteins, and thus its specificity is low because it also reacts with SARS-CoV. Our system therefore provides overwhelming advantages in relation to cost, detection specificity, and simplicity, without requiring a special instrument.

Even though SARS-CoV-2 spike proteins could be detected at the attomole level in the present study, it is important to demonstrate that our system can detect live SARS-CoV-2 in actual specimens obtained from COVID-19-positive patients. Experiments using live SARS-CoV-2 must be performed in biosafety level 3 (BSL3) facilities. We do not have such facilities at Waseda University or TAUNS Laboratories Inc., and the BSL3 facilities in public institutions are currently not available because they are being fully utilized. We, however, notice that the virus envelope is destroyed by detergent [27], and a detergent solution can easily separate spike proteins from viruses. Thus, by using the ultrasensitive system to detect the spike proteins of detergent-solubilized SARS-CoV-2, this antigen test can be used to diagnose COVID-19. Furthermore, we should note that the SARS-CoV-2 spike proteins are highly conserved among different isolates [28].

Our best detection sensitivity data were obtained from an ultrasensitive ELISA combined with 60 min of thio-NAD cycling. There is a trade-off between optimizing detection sensitivity and minimizing detection time. That is, decreasing the detection time decreases the detection sensitivity. As shown in Figure 3, the LOD of the 10-min detection assay was on the order of 10^−17^ moles/assay. We are currently working toward testing our system in a BSL3 facility to detect SARS-CoV-2 spike proteins from specimens obtained from COVID-19 patients. At that time, we will determine the optimal cycling time of our system for detecting the antigen at clinically relevant levels.

## 5. Conclusions

To the best of our knowledge, the newly developed system described here is the first ultrasensitive antigen test for SARS-CoV-2 spike proteins, and can be performed with an easy-to-use microplate reader. Our antigen test is easy, rapid, reasonably priced, specific, and ultrasensitive, allowing for simultaneous multiple measurements of SARS-CoV-2 and thus applicable for diagnosing COVID-19. Although our system requires only a microplate reader, we are currently working on producing an automated apparatus for using our system. Our system can be used for rapid virus detection to implement quarantine policies, such as in airports.

## Abbreviations

3α-HSD	3α-hydroxysteroid dehydrogenase
ACE2	angiotensin-converting enzyme 2
ALP	alkaline phosphatase
BSA	bovine serum albumin
CLEIA	chemiluminescent enzyme immunoassay
COVID-19	coronavirus disease 2019
CV	coefficient of variation
ELISA	enzyme-linked immunosorbent assay
LOD	limit of detection
LOQ	limit of quantitation
PCR	polymerase chain reaction
SARS-CoV-2	severe acute respiratory syndrome coronavirus 2
TBS	Tris-buffered saline
thio-NAD	thio-nicotinamide adenine dinucleotide
WHO	World Health Organization
